# Analysis of γ′ Precipitates, Carbides and Nano-Borides in Heat-Treated Ni-Based Superalloy Using SEM, STEM-EDX, and HRSTEM

**DOI:** 10.3390/ma13194452

**Published:** 2020-10-08

**Authors:** Łukasz Rakoczy, Bogdan Rutkowski, Małgorzata Grudzień-Rakoczy, Rafał Cygan, Wiktoria Ratuszek, Anna Zielińska-Lipiec

**Affiliations:** 1 Faculty of Metals Engineering and Industrial Computer Science, AGH University of Science and Technology, Mickiewicza 30, 30-059 Kraków, Poland; rutkowsk@agh.edu.pl (B.R.); ratuszek@agh.edu.pl (W.R.); alipiec@agh.edu.pl (A.Z.-L.); 2 Łukasiewicz Research Network-Kraków Institute of Technology, Zakopiańska 73, 30-418 Kraków, Poland; malgorzata.grudzien@kit.lukasiewicz.gov.pl; 3 Investment Casting Division, Consolidated Precision Products Corporation, Hetmańska 120, 35-078 Rzeszów, Poland; Rafal.Cygan@cppcorp.com

**Keywords:** superalloy, HRSTEM, STEM-EDX, M_23_C_6_, nano-borides

## Abstract

The microstructure of a René 108 Ni-based superalloy was systematically investigated by X-ray diffraction, light microscopy, energy-dispersive X-ray spectroscopy, and electron microscopy techniques. The material was investment cast in a vacuum and then solution treated (1200 °C-2h) and aged (900 °C-8h). The γ matrix is mainly strengthened by the ordered L1_2_ γ′ phase, with the mean γ/γ′ misfit, δ, +0.6%. The typical dendritic microstructure with considerable microsegregation of the alloying elements is revealed. Dendritic regions consist of secondary and tertiary γ′ precipitates. At the interface of the matrix with secondary γ′ precipitates, nano M_5_B_3_ borides are present. In the interdendritic spaces additionally primary γ′ precipitates, MC and nano M_23_C_6_ carbides were detected. The γ′ precipitates are enriched in Al, Ta, Ti, and Hf, while channels of the matrix in Cr and Co. The highest summary concentration of γ′-formers occurs in coarse γ′ surrounding MC carbides. Borides M_5_B_3_ contain mostly W, Cr and Mo. All of MC carbides are enriched strongly in Hf and Ta, with the concentration relationship between these and other strong carbide formers depending on the precipitate’s morphology. The nano M_23_C_6_ carbides enriched in Cr have been formed as a consequence of phase transformation MC + γ → M_23_C_6_ + γ′ during the ageing treatment.

## 1. Introduction

Ni-based alloys used for working at high homologous temperatures are the most advanced metallic construction materials. The unique combination of high strength and excellent oxidation resistance makes it an irreplaceable group of materials in aerospace, power, and nuclear industries. The usefulness of Ni-based superalloys during long-term service at harsh conditions depends strongly on the alloying elements, their concentrations, and the morphology of the main strengthening phases [1,2,3,4]. The development of the first Ni-based superalloys began over 100 years ago. It happened around 1918 with the patenting of the Ni-20Cr alloy (Nichrome) [5,6]. It became a progenitor for the next superalloys of the Nimonic and Inconel series [7]. In 1929, Bedford, Pilling, and Merica presented the results of the study on the influence of Al and Ti on the mechanical properties of the Nichrome superalloy [1,5]. They noticed that the addition of these elements significantly increases creep resistance. Despite difficulties in observing fine precipitates responsible for the strengthening, several subsequent Ni-based superalloys were developed [8]. In the following years, some of the nickel content was replaced with cobalt, thus increasing the high-temperature oxidation resistance. One of the main representatives of Ni-Cr-Co alloys was Nimonic 90 with 20% of cobalt [9]. In the 1930’s the gas turbine design by Elling had an operating temperature which did not exceed 550 °C [10]. There was no huge demand for Ni-based superalloys because the austenitic stainless steels could still work safely at this temperature. Several years later engines constructed by Heinkel and Whittle led to breakthrough in the Ni-superalloys’ development because the service temperature reached 780 °C [11,12]. Conventional stainless steels were not able to meet the growing requirements [13]. During the Second World War superalloys were therefore modified by introducing even higher amounts of aluminium and titanium. Meanwhile, at the end of the 1940s, it was noticed that the additions of refractory elements, like Mo, contribute to a significant solution and precipitation strengthening with carbides. In the 1940s, engine components were manufactured by forging and air casting and so the strength and metallurgical purity were not the highest. The introduction of vacuum casting technology in 1952 was considered to be the most essential step towards improving the mechanical properties and purity of both cast and wrought superalloys [4,14]. Before the advent of vacuum melting and casting techniques, cast superalloys did not gain wide acceptance for a turbine blade application. One of the first alloys to be vacuum cast was Inconel 713C and this became widely used in the aerospace industry. The high carbon content contributed to excessive primary carbides, poor ductility at low temperatures and problems with integrally bladed disc castings. To overcome this issue, a low carbon version of the alloy was made and is also widely used for turbine blades [14,15,16]. In the late 1950s, H.L. Eiselstein developed Inconel 718 precipitation strengthened by intermetallic phase γ″ (Ni_3_Nb), which, along with the Waspaloy, was quickly put into continuous production [4,17]. In the 1960s, it began to be seen that chromium could be partially replaced to achieve even higher creep resistance. However, limiting its concentration sensitized superalloys to high-temperature corrosion and so more attention was paid to its influence on microstructure and properties [4,18]. Numerous chemical composition modifications broadened the knowledge of metallurgists and allowed to develop new grades of superalloys, but also this was associated with design faults. Excessive amounts of alloying elements led to the formation of detrimental topologically closed phases (TCP) [19]. These undesired results indicated that in such complex materials design, it is necessary to use additional tools, computer software [20]. In the late 1960s, the strengthening of superalloys by only increasing Al and Ti became impractical. Taking into account only weight fraction, they were not the dominant alloying elements. Their too high concentration prevents the production of complex castings due to brittleness and so Al + Ti concentration usually does not exceed 8% [21,22]. Thanks to lowering of the Ti concentration, the castability was considerably improved, which consequently led to design of the new superalloys with low Ti/Al ratio [23]. Additionally, the Mo and Ta concentrations were increased in order to strengthen the γ-matrix. The proposal of molybdenum replacement by tungsten was adopted during the development of the MAR M200 by Martin Metals Company. Too high creep rate led to premature failure, and in response to this result, the superalloy was modified by hafnium, and so the MAR M002 was introduced [14,24]. Hf leads to fine and stable MC carbides’ formation. This contributed to the modification of many previously developed superalloys like Inconel 713C (to MM-004) and B1900 (to MM-007) [25,26]. Another, approach to overcoming the intermediate temperature ductility problem in cast superalloys has been the development of the so-called BC superalloys [14,27]. Maxwell [28] found that the high boron content (0.1–0.16%) with a low carbon concentration guarantees the improvement of the creep resistance of MAR M200 superalloy at 760 °C, without reducing other properties. Quichou [29] confirmed that BC superalloys were characterized by better castability properties than IN792, IN792+Hf, and IN100. This impact was the result of borides’ formation in the grain boundaries. A similar effect was obtained in many other superalloys, thanks to which boron is still an essential alloying element [30,31,32]. In the following years, the design of polycrystalline alloys assumed the use of all previously known mechanisms. After several decades, the archetype of the superalloys, two-component Nichrome, was modified to such an extent that the alloying elements exceeded twelve. The best examples of this are the microstructurally complex superalloys as MAR-M247 and Inconel 792+Hf [33,34,35]. 

To understand the behaviour of the especially complex Ni-based superalloys at high-homologous temperatures it is necessary to characterize their microstructures in fully heat-treatment condition. Detection and characterization of precipitates, even nano-precipitates, is essential in the study of creep and fatigue degradation. Without the knowledge about the phase composition of material, it is problematic to predict the microstructure evolution during long-term service. The main aim of this work was to perform the comprehensive characterization of equiaxed Ni-based superalloy René 108 from micro- to atomic-scale resolution by using analytical microscopy techniques.

## 2. Experimental Procedure

The experimental material was a René 108 Ni-based superalloy casting. It was heat treated in a vacuum with the following parameters: (a) solution: 1200 °C-2h; (b) ageing: 900 °C-8h. Chemical composition designated by optical emission spectroscopy (OES) is presented in Table 1.

Microstructural observations and analyses were performed using: thermodynamic simulations, X-ray diffraction (XRD), light microscopy (LM), scanning electron microscopy (SEM), scanning-transmission electron microscopy (STEM), high-resolution scanning-transmission electron microscopy (HRSTEM), dispersive X-ray spectroscopy (EDX). Thermo-Calc software (ThermoCalc Software AB, version 2020a, Solna, Sweden) (database TCNI6:Ni-Alloys) was applied to calculate the maximum solubility of selected alloying elements (Cr, Co, W, Al, Ti, Ta, Hf, Mo, B, C) in the Ni-matrix at 1200 °C and 900 °C. XRD study was on a Siemens/Bruker D5005 diffractometer using Co K_α_ (λ = 1.79 Å) radiation source in Bragg-Brentano geometry. The samples were wire cut from the part into a plate with dimensions 20×20×10 mm and then were ground and polished on a diamond suspension. The XRD patterns were acquired at a scan rate of 0.04 °/s with 2θ (Bragg angle) and a scan range from 20° to 130°. The angles were read off from the positions of the peaks on a diffractogram and the interplanar spacings d_khl_ were calculated using the Bragg-Wulff equation (Equation (1)). Deconvolution for the double-peaks was performed by the least-square method. Based on the computed lattice a_γ_ and a_γ′_ parameters (Equation (2)), the γ/γ′ misfit coefficient (Equation (3)) was determined.

(1) $$d_{hkl}=\frac{\lambda }{2\sin\theta }$$

(2) $$a=\frac{\lambda \sqrt{h^{2}+k^{2}+l^{2}}}{2\sin\theta }$$

(3) $$\delta =\frac{2\left(a_{\gamma^{\prime }}-a_{\gamma }\right)}{a_{\gamma^{\prime }}+a_{\gamma }}$$

The samples (30×30×30 mm) for LM and SEM observations, cut from the investment casting, were ground, polished (diamond suspensions: 3 μm, 1 μm; colloidal silica: 0.25 μm), and electrochemically etched in 10% oxalic acid (C_2_H_2_O_4_). Initial characterization of morphology and distribution of precipitates was carried out on a Leica light microscope (Leica Microsystems, Wetzlar, Germany). For SEM-EDX analyses, 20 kV accelerating voltage was set on a Phenom XL (Phenom-World, Eindhoven, Netherlands) apparatus equipped with Energy Dispersive X-ray Spectroscopy detector. Segregation coefficient (concentration of alloying element “i” in the center of dendrite arm divided by concentration in the interdendritic space, determined by SEM-EDX, was calculated in accordance with Equation (4), based on the 20 measurements in various locations (each area 10 × 10 µm). Quantitative chemical analysis was carried out using the ZAF correction.

(4) $$k^{i}=\frac{C_{D}^{i}}{C_{ID}^{i}}$$

where:

$C_{D}^{i}$—concentration of “*i*” alloying element in dendritic region, at%
$C_{ID}^{i}\;$—concentration of “*i*” alloying element in interdendritic space, at%

Image analysis of the precipitates was performed by ImageJ commercial software (National Institutes of Health and the Laboratory for Optical and Computational Instrumentation, University of Wisconsin, 1.51j8, Madison, WI, USA). The relative volume of carbides and γ′ precipitates was determined by the planimetric method. According to the Cavalieri-Hacquert principle, the estimator of the relative volume occupied by a given component of the alloy structure is the fraction of the area occupied by this component on the unit plane of the sample:

(5) $$V_{v}=A_{A}$$

(6) $$A_{A}=\left(\frac{\Sigma A_{i}}{A}\right)*100\%$$

where:

$V_{v}$—total volume of the phase object per unit volume of the alloy, μm^3^/μm^3^
$A_{A}$—total field flat sections on the individual phase of the image per unit area, μm^2^/μm^2^
$A_{i}$—total field of flat sections on the individual i-phase, μm^2^
*A*—total image area, μm^2^

For the calculation of carbides’ volume fraction, the 5 images captured by LM under magnification x100 were used (area 1.18 mm^2^). In the case of γ′ 10 images were used at ×20000 magnification (area 179.8 μm^2^). All images were put to binarisation and a despeckle filter, which removed noise without blurring edges. The area (A) and perimeter (P) of each γ′ precipitate (from the area range 0.06–5.0 μm^2^) were measured to calculate the distribution of γ′ shape factor ξ = (4Π*A)/(P^2^). The mean size of the γ′ was represented by the equivalent radius for the area. Stereological analysis for γ′ precipitates was carried for more than 1650 precipitates in a dendritic region. Specimens for STEM and HRSTEM investigation were prepared by ion polishing. Slices were first ground mechanically to a thickness of about 0.05 mm, and then 3 mm discs were punched from these ground samples and dimpled using a Gatan Dimple Grinder (Gatan, Inc., Pleasanton, CA, USA) on each side. The last step was thinning by Ar^+^ ion beam (PIPS of Gatan). Before loading into the microscope, the thin foils were cleaned by a plasma cleaner for removing surface contamination. Investigations were performed using a probe Cs-corrected FEI Titan Cubed G-2 60-300 (Thermo Fisher Scientific, Eindhoven, Netherlands) with ChemiSTEM system operating at 300 kV. Images in atomic scale resolution were analyzed by Fast Fourier Transformation (FFT) to obtain position of diffraction peaks. The patterns were solved with JEMS software (JEMS-SWISS, Jongny, Switzerland).

## 3. Results and Discussion 

### 3.1. Calculation of the Alloying Elements Solubility in Nickel by Thermo-Calc 

Table 2 summarizes atomic radius [36] and solubility data for various elements in Ni at 1200 °C and 900 °C calculated by thermodynamic simulations with Thermo-Calc. It is helpful information in the phase analysis of Ni-based superalloy by advanced electron microscopy. Co and Ni exhibit complete solid solution in the face-centered cubic structure. Besides these, the elements characterized by the highest solubility in the Ni matrix include Cr and Mo. At 1200 °C it is 49.53 at% and 25.97 at%, respectively. As the temperature drops to 900 °C, the solubility of Cr and Mo in γ matrix decreases, however, these values are still high. The main strengthening phase in cast nickel superalloys is γ′ with the stoichiometric formula Ni_3_Al. The solubility of Al in Ni for cooling 1200 °C → 900 °C changes from 18.38 at% to 14.38 at%, while Al position in the unit cell can be replaced by other elements as Ti and Ta. Hf is characterized by remarkably low solubility in nickel, which drops from 1.24 at% at 1200 °C to just 0.26 at% at 900 °C. In contrast, carbon causes Hf, as also Ta, Ti, and W to form carbides. From all alloying elements, boron has the lowest solubility in Ni, both at 1200 °C (0.36 at%) and 900 °C (0.019 at%). If its content in the material exceeds the maximum solubility in Ni, it creates favorable conditions for borides’ formation. To summarise, the limited solubility in Ni of several alloying elements results in strengthening phases in the René 108 microstructure, i.e., intermetallic phases, carbides and borides.

### 3.2. Characterization of Phase Compositions and γ/γ′ Lattice Misfit by XRD 

**Figure 1:**
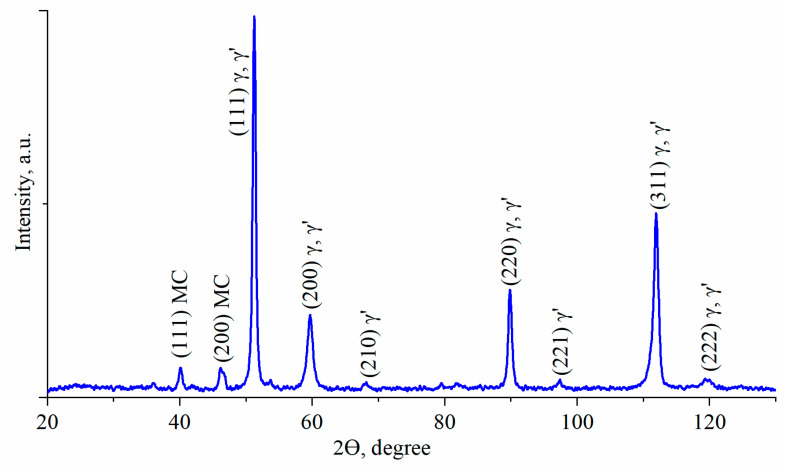
XRD pattern of René 108 (Co K_α_ radiation), λ = 1.79 Å.

The XRD pattern for heat-treated René 108 is presented in Figure 1. It reveals peaks of three phases: γ matrix, intermetallic phase γ′ and MC carbides. In Table 3, the interplanar spacing d_hkl_ and lattice parameters of detected phases are shown as measured on the XRD pattern. The mean lattice parameter of the matrix and precipitates determined using analyzed reflections were 3.59 Å (±0.01 Å) and 3.60 Å (±0.02 Å), respectively. The mean misfit coefficient calculated by Equation. (3) based on all double peaks is positive, δ = +0.6% (±0.5%). Note that the misfit value is an average value because of the relatively high level of alloying elements segregating in the dendritic structure of equiaxed superalloys. Lattice misfit is related to the morphology of precipitates. Superalloys with spherical precipitates are characterized by misfit ±0–0.2%, and with cubic ±0.5–1% and plate-like precipitates > ±1.25% [37]. Lattice misfit has long been associated with alloy strengthening, and so low misfit is preferred to withstand coarsening. Typical heat treatment (solution + ageing) is generally carried out to favour cubic morphology of γ′ precipitates.

### 3.3. Microstructure of René 108 Superalloy by LM and SEM

**Figure 2:**
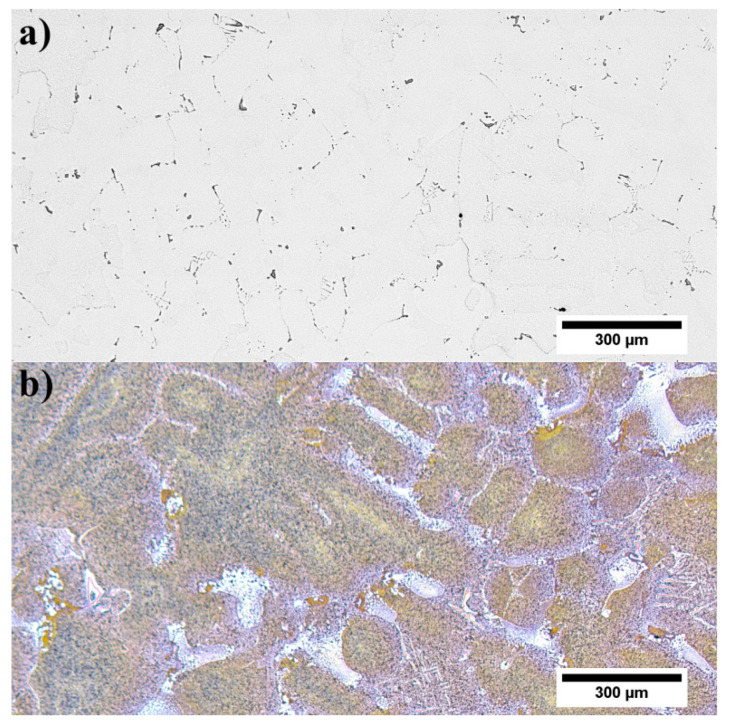
Microstructure of René 108: **a**) unetched; **b**) etched, light microscopy (LM).

The unetched microstructure of the superalloy reveals the carbides’ distribution and their morphology (Figure 2a). Their volume fraction is 0.88% (±0.16%). The high standard deviation resulted from their uneven distribution in the casting volume. Carbides are characterized by a complex morphology, namely small regular blocks, elongated parallelograms with smooth edges, and “Chinese script”. The last one usually consisted of three separate parts: the central core terminated with an angular head and perpendicularly arranged elongated arms. The core and arms were made of fine, irregular carbides, unlike thick plates and rods. Etching revealed the dendritic structure of the casting and significant local microsegregation of microstructural components (Figure 2b). Despite the heat treatment (solution + ageing), the microstructure is still very heterogeneous. The high content of alloying elements, with their limited solubility in the γ matrix, has led to the formation of numerous constituents. The dendritic areas are characterized by a relatively homogeneous microstructure with γ′ precipitates of various sizes. In the interdendritic spaces, carbides and eutectic islands γ-γ′ are additionally present.

**Figure 3:**
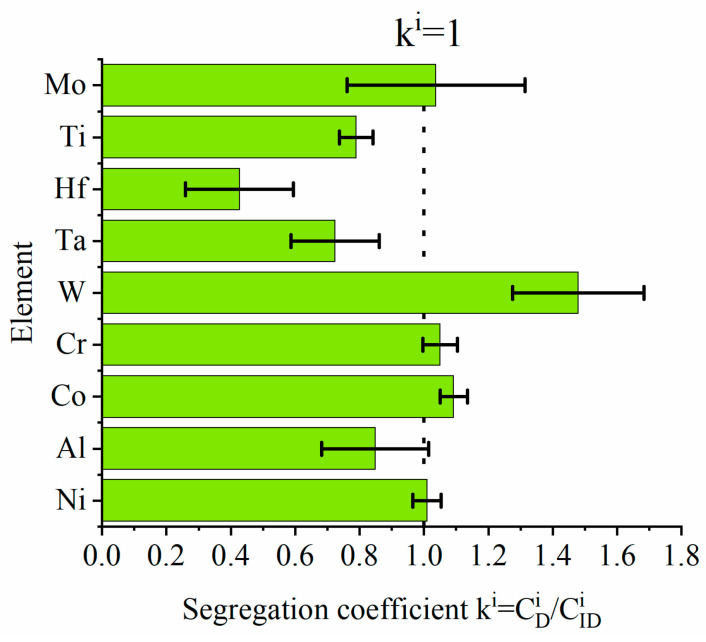
Segregation coefficient $k^{i}=\frac{C_{D}^{i}}{C_{\mathrm{ID}}^{i}}$ of selected alloying elements.

Significant differences in the microstructure of dendritic areas in relation to interdendritic spaces indicate that segregation of the alloying elements is present. To show this quantitatively, SEM-EDX measurements in these both regions were carried out. The data were used to calculate the segregation coefficient $k^{i}=\frac{C_{D}^{i}}{C_{\mathrm{ID}}^{i}}$ of each alloying element. The ratio of the concentration of an alloying element in the centerline of dendrite arms to the concentration in the interdendritic spaces was calculated and is presented in Figure 3. Results confirm the irregular distribution of elements. The k^i^ values, when lower than one, indicate that the element segregates to interdendritic spaces. When k^i^ > 1, the element is more concentrated in the dendritic regions. Elements that strongly segregate to interdendritic spaces are Hf, Ta, Ti, and Al. When k^i^ values are below one, corresponding to W, Co, and Cr, these elements are more concentrated in dendrites. The most uniform distribution is observed for Ni and Mo.

**Figure 4:**
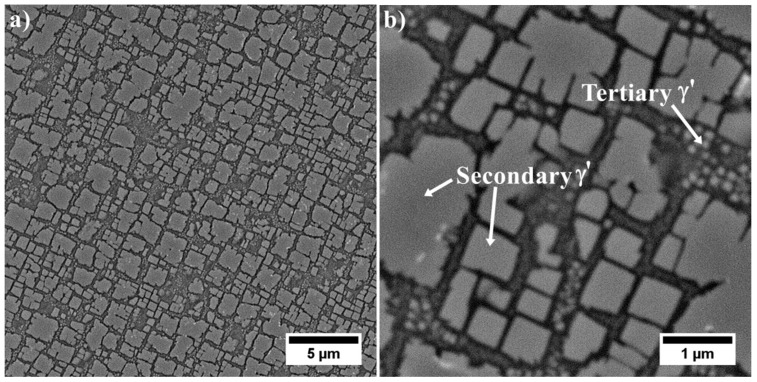
Microstructure of precipitates in a dendrite arm: (**a**) secondary γ′; (**b**) secondary and fine tertiary γ′.

**Figure 5:**
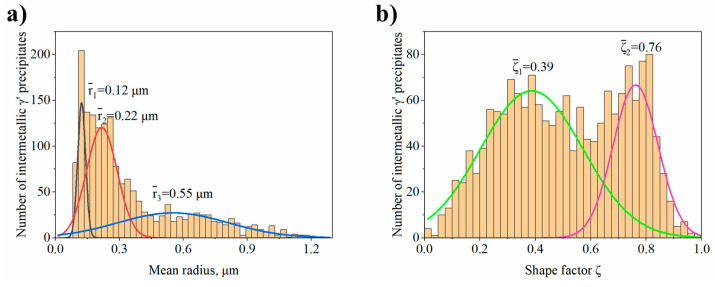
(**a**) the γ′ mean radius distribution; (**b**) shape coefficient ζ distribution.

Using the backscattered detector, SEM images with a strong dependence on the average atomic number Z were obtained. The microstructure of the γ′ in dendritic regions is presented in Figure 4. Large secondary γ′ precipitates are characterised by a complex morphology. Locally at their edges there are single precipitates. During homogenization, secondary γ′ precipitates were dissolving in the matrix. Some of those that were not completely dissolved, coagulated, and coalesced. During cooling, they became nucleation sites for re-precipitating from the supersaturated γ solution. The misfit stresses between the γ and γ′ phases promoted nucleation in the stress fields of subsequent precipitates in these sites. Finer secondary γ′ precipitates had a cubic-like morphology. The differences in their morphology are due to the various temperatures and time ranges in which they formed, as well as the low coagulation rate during ageing. Inside the γ channels, between secondary γ′ precipitates, spherical tertiary γ′ precipitates were revealed, formed independently from the supersaturated matrix during cooling after the ageing treatment. The volume fraction of the γ′ precipitates within the dendrites calculated using SEM-BSE images is 54.99% (±3.65%). The γ′ mean radius distribution presented in Figure 5a clearly reveals three classes which belong to large and fine secondary γ′ and also tertiary γ′. Based on the area and perimeter of each precipitate, its shape coefficient was calculated. All obtained values are summarised in Figure 5b. Two curves are fitted to the distribution. The green one corresponds to the large secondary γ′ precipitates and the second pink curve to cubic secondary and spherical tertiary γ′. According to the shape factor equation ξ = (4Π*Area)/(Perimeter^2^), the cubic precipitates have a coefficient of ζ 0.785, while the spherical ζ of 1.0. Variation in nature (size, shape, and volume fraction) of γʹ precipitates is observed among others due to the effects of microsegregation and local cooling rates during casting solidification.

**Figure 6:**
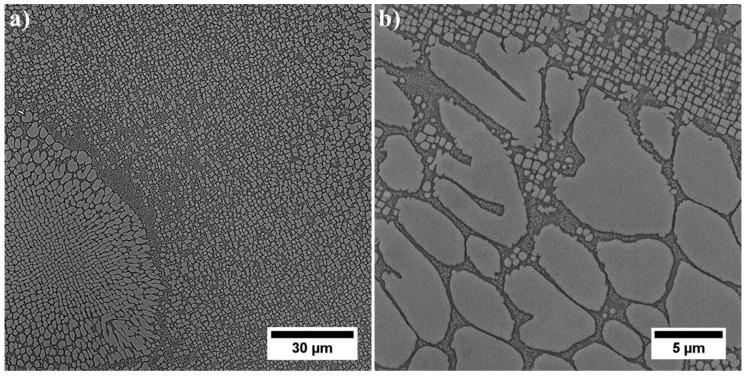
Microstructure of γ′ precipitates in interdendritic space: (**a**) difference in size and morphology of precipitates; (**b**) large primary γ′ precipitates.

In the interdendritic spaces, the primary, secondary, and tertiary γ′ precipitates are characterized by much greater variability in distribution and morphology (Figure 6). These factors do not allow us to compare effectively stereological parameters with precipitates inside the dendrites. The primary γ′ precipitates created at the end of casting’s solidification, through the L→ γ + γ′ phase transformation have an especially complex morphology. It is suggested that this phase transformation was monovariant, namely progressing in the temperature range. The dissolution of large eutectics is challenging during industrial solid solution treatment, while increasing the solution temperature may lead to undesirable incipient melting. The eutectic γ-γ′ islands are an unwanted component in superalloys, but their complete dissolution in the matrix during solution treatment is not possible. However, the high solution temperature changed the thermodynamic conditions and lead to partial dissolution, which consequently influenced the differentiation of the size of the secondary precipitates of the γ′ phase around the eutectic γ-γ′ islands.

**Figure 7:**
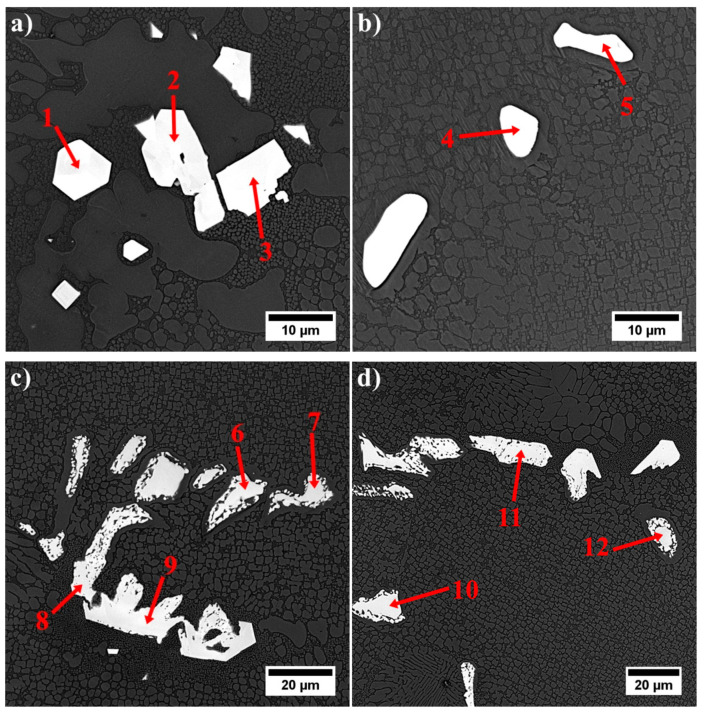
Microstructure of MC carbides and location of SEM-EDX analysis points: (**a**) blocky carbides and points no. 1–3; (**b,c**) carbides surrounded by the coarse γ′ and points no. 4–9; (**d**) morphologically complex carbides and points no. 10–12.

**Figure 8:**
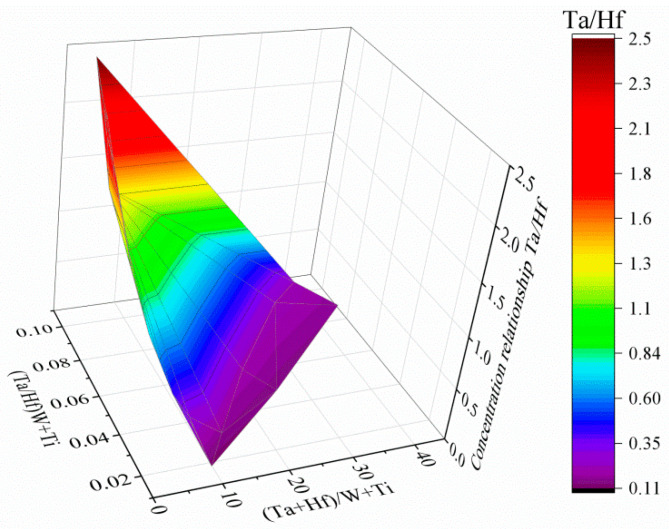
Graphical representation of selected concentration relationships between alloying elements in carbides. Based on the SEM-EDX point analysis.

MC precipitates appear as “white” precipitates and are concentrated in the interdendritic areas. For all of them, the Z-contrast is almost the same. Semi-quantitative SEM-EDX analyses of MC carbides were carried out to show the relationships between carbide formers, because it is widely known that carbides of metals belonging to the same class show considerable inter-solubility. Results from selected locations are presented in Figure 7 and Table 4. In chemical compositions of all carbides, strong carbide formers dominate, especially hafnium and tantalum. The mutual relation between these elements change, depending on the shape and size of a precipitate. The following relationships were calculated between the elements with the highest affinity for carbon: Ta/Hf, (Ta + Hf)/(W + Ti) and (Ta/Hf)/(W + Ti). Blocky shaped carbides with sharp edges located in the close vicinity of eutectic γ-γ′ islands are the only ones characterized by a low Ta/Hf ratio and a very high (Ta + Hf)/(W + Ti) (more than a dozen). The carbides surrounded by the coarse γ′ phase had the Ta/Hf and (Ta + Hf)/(W + Ti) coefficients in the range 2.0–3.0 and the (Ta/Hf)/(W + Ti) ≤ 0.1. The morphology of the last group of carbides is similar to the previously described, with one general difference, lack of surrounding coarse γ′. Relation Ta/Hf is 0.29–0.75, whereas (Ta + Hf)/(W + Ti) is in the range 4.75–8.06 and the (Ta/Hf)/(W + Ti) ≤ 0.05. An additional 40 measurements were performed to present graphically the concentration relationships between strong carbide formers in the carbides’ composition (Figure 8). Relationships in the corners of the graph correspond with these in Table 4. 

**Figure 9:**
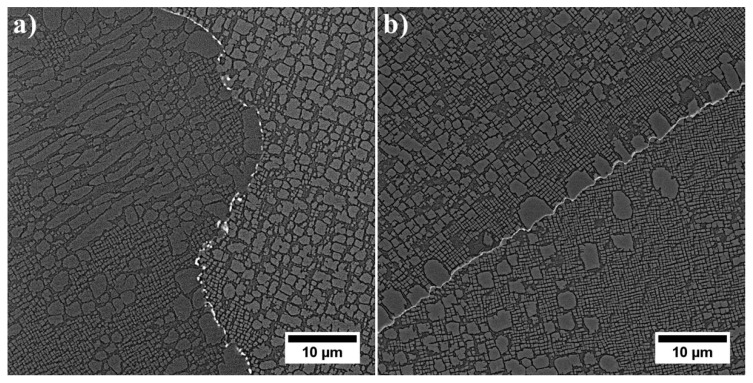

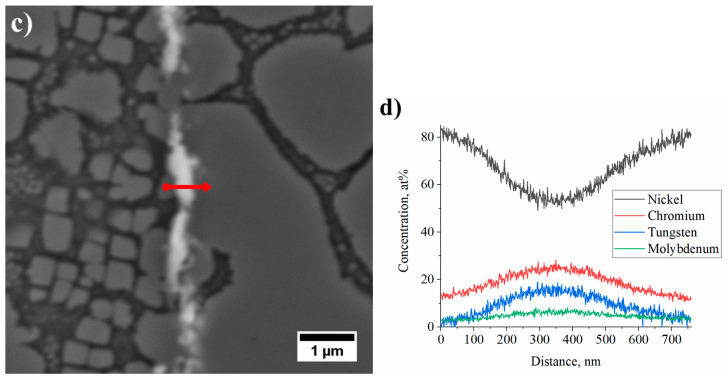
(**a,b**) microstructure of precipitates at the grain boundaries; (**c**) location of linear SEM-EDX analysis; (**d**) distribution of selected alloying elements at a grain boundary.

At a grain boundary, continuous or semi-continuous layers of fine precipitates with irregular morphology are observed (Figure 9a,b). Crosswise to the grain boundary, a linear analysis of the distribution of selected alloying elements in one of these precipitates was performed (Figure 9c,d). Enrichment in chromium and tungsten is revealed, and also slightly increased Mo content. Alloying elements like Cr, W, and Mo are generally known to have a high affinity for boron/carbon, and the solid solubility of boron/carbon in the γ phase is very low (as presented in Table 2). It is likely that borides and/or secondary carbides can easily form at the grain boundaries in René 108.

### 3.4. Characterization of Strengthening Phases by STEM and HRSTEM

#### 3.4.1. Dendritic Regions

**Figure 10:**
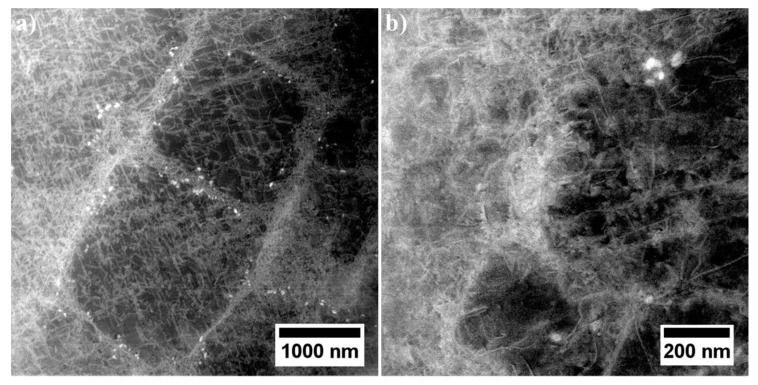
Microstructure of secondary γ′ with nano-precipitates at the γ/γ′ interface: (**a**) cubic-shaped γ′ precipitates; (**b**) complex-shaped γ′ precipitate, STEM-HAADF.

The γ phase constituting the matrix of René 108 superalloy is strengthened mainly with the γ′ phase. In the narrow channels of the γ matrix, between the large secondary γ′ precipitates, nanometric tertiary γ′ precipitates are present (Figure 10). The interfaces γ/γ′ are additionally occupied by bright nano-precipitates. The degree of strengthening and mechanical properties depends on the chemical composition of the precipitates and the matrix, as well as the mutual crystallographic relationship, which was also analysed.

**Figure 11:**
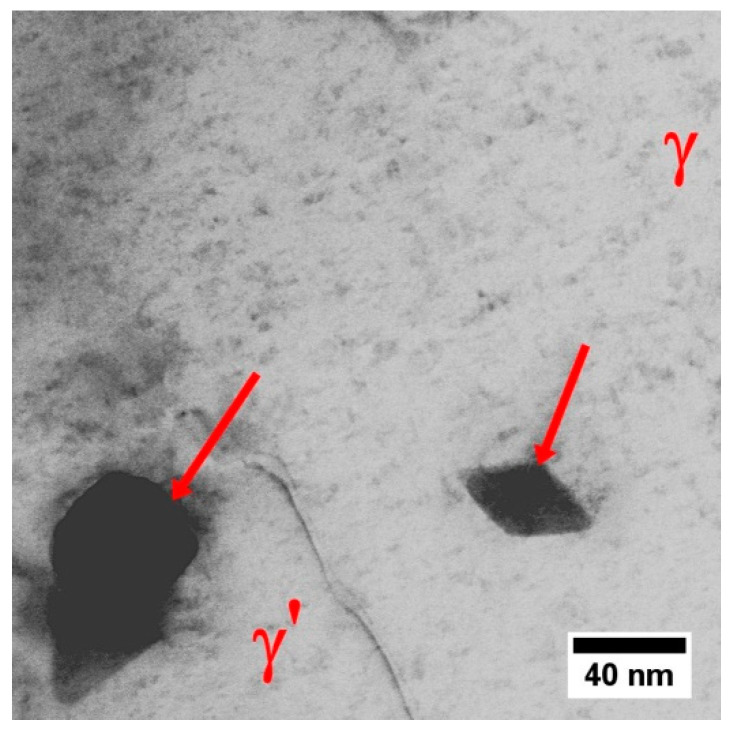
Nano-precipitates in dendritic regions, STEM-BF.

The STEM-HAADF microstructure of dendritic region contains nano-precipitates at the interfaces γ/γ′. Bright contrast indicates that they consist mainly of elements with higher atomic numbers Z than those in phases γ and γ′ (Figure 10a). During observations of thin foils at higher magnification, it was confirmed that these precipitates differ in morphology (Figure 11), with the one precipitate type being polygon-shaped with a 50 nm diameter, and the other being diamond-shaped (50 × 25 nm). 

**Figure 12:**
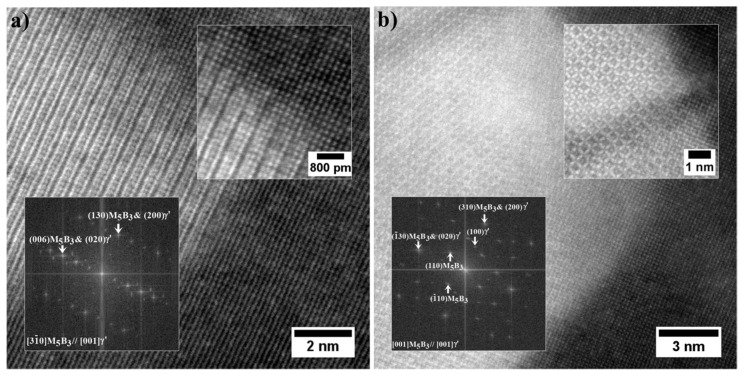
Nanostructure of the M_5_B_3_ boride with corresponding Fast Fourier Transformation (FFT) image: (**a**) Zone axis [$3\bar{1}0]$M_5_B_3_; (**b**) Zone axis [001]M_5_B_3_, HRSTEM-HAADF.

Nanostructure of the precipitates and their interfaces with the γ′ is shown in Figure 12. Based on the FFT image analysis, both precipitates are confirmed to be M_5_B_3_ borides. This phase is characterized by a body-centered tetragonal crystal structure with the space group I4/mcm and lattice parameters of a = 5.46 Å and c = 10.64 Å [38]. It has a D8_1_-type structure (Strukturbericht notation) based on the information of atomic occupations in M_5_B_3_ phase. The atomic structure of boride obtained along the four-fold [001]M_5_B_3_ direction of the M_5_B_3_ phase is presented in Figure 12b. Although B atoms are invisible in the HAADF images due to their weak scattering ability, the structural characteristics of the M_5_B_3_ phase can be displayed. Imaging M_5_B_3_ along [001] zone axis reveals only one type of Wyckoff site. The relationship the γ/γ′ and M_5_B_3_ boride phases can be written as: [001]γ, γ′//[001]M_5_B_3_; (200)γ, γ′//(130)M_5_B_3_ and [001]γ, γ′//[$3\bar{1}0]$M_5_B_3_; (020)γ, γ′//(006)M_5_B_3_. The orientation relationship between M_5_B_3_ and γ/γ′ has also been investigated by other authors. Han [39] and Sheng [40] observed the relationship [001]γ//[001]M_5_B_3_ in model superalloys, while Zhang [41], based on René 80 superalloy study, indicated that [010]γ//[130]M_5_B_3_ and [001]γ//[001]M_5_B_3_.

**Figure 13:**
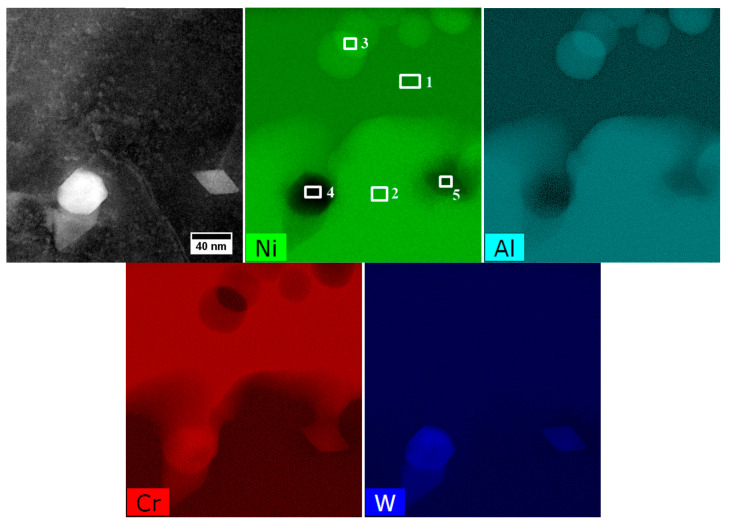
Distribution of selected alloying elements in a dendritic region, STEM-EDX.

The STEM-EDX mapping of alloying elements covering the γ matrix channel and γ′ precipitates with nano-precipitates at the γ/γ′ interface is presented in Figure 13. Additionally, the quantitative analysis was carried out in 5 regions (Table 5). It was found that the main elements strengthening the γ matrix (precipitates-free zone, PFZ) are Cr and Co, with concentrations of over 35 at%. Among the elements which usually form (with Ni) the γ′ precipitates are Al, Ti, Ta, and Hf. Their total concentration in the secondary γ′ phase is 17 at%, while in the tertiary is slightly lower, 15.4 at%.

**Figure 14:**
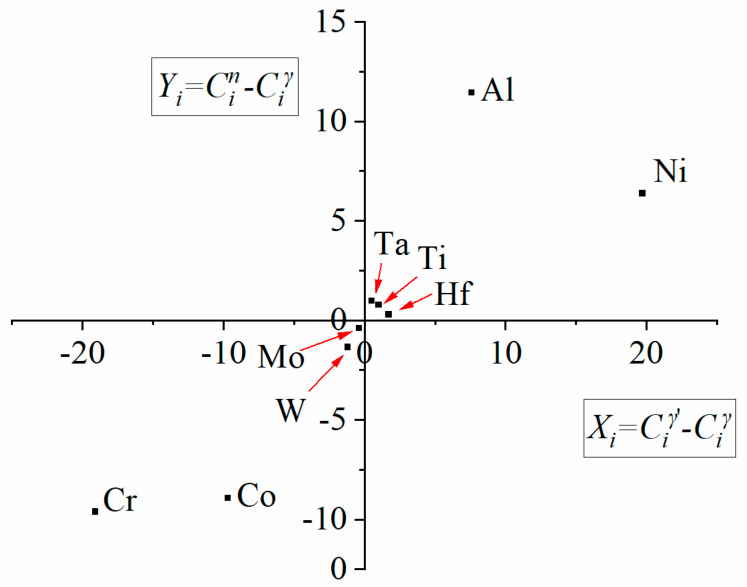
Plot $C_{i}^{n}-C_{i}^{\gamma }$ vs. $C_{i}^{\gamma^{\prime }}-C_{i}^{\gamma }$ calculated based on the composition of the bulk superalloy, secondary γ′ and the matrix.

The mapping and analysis in selected areas confirmed that the partitioning of the alloying elements occurs between γ and γ′ phases. The additional chemical composition measurements by STEM-EDX of γ matrix and secondary γ′ phases were performed in 10 other locations to obtain better statistics; the results are presented in Figure 14. The concentration of selected alloying element (*i* = Ni, Cr, Co, etc.) in γ and secondary γ′ is presented as $C_{i}^{\gamma }$ and $C_{i}^{\gamma^{\prime }}$, respectively. $C_{i}^{n}$ is the nominal concentration of an element in the bulk superalloy. The elements most strongly segregating to the intermetallic γ′ precipitates, i.e., those placed in the first quarter, are Ni, Al, Ta, Ti, and Hf. Alloying elements in the γʹ phase alter the formation energies of anti-phase boundaries (APBs), super lattice intrinsic stacking faults (SISFs), and complex stacking faults (CSFs), and may also directly influence the strength and plasticity of the γʹ phase. The strengthening effect of the intermetallic γ′ precipitates could be improved by the presence of elements like Ta and Ti because Ta-Ta and Ti-Ti bonds have higher energies than Al-Al bond [42].

**Figure 15:**
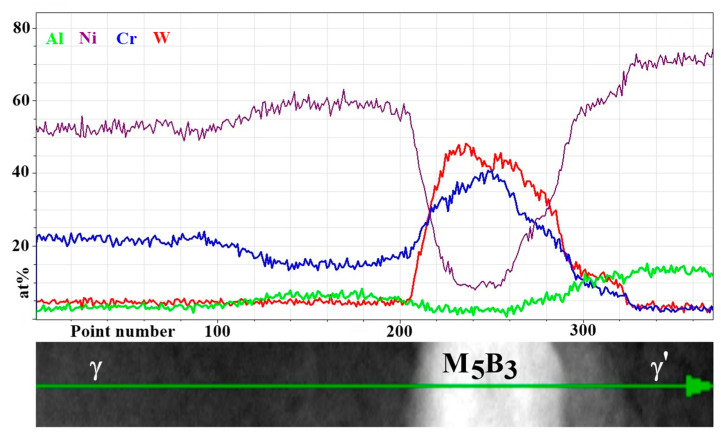
Distribution of selected alloying elements in γ, M_5_B_3_ and γ′ phases, STEM-EDX.

In accord with results of STEM-EDX analyses, in areas no. 4 and 5 (Table 5) borides are enriched in W, Cr, and Mo. Therefore they can be described by the formula (W, Cr, Mo)_5_B_3_. The concentration relationship of the two dominant W/Cr elements in the examined areas is 1.2, and 1.4, respectively, and the W/Mo relationship is 8.1 and 9.0. These relations are therefore very similar, and the differences in the relative concentration results from the small thickness of the precipitate with a diamond shape. The distribution of selected elements presented in Figure 15 shows the sharp increase of tungsten concentration at the interface γ/M_5_B_3_. In the case of Cr, the concentration growth is more smoother and occurs over a longer distance.

#### 3.4.2. Interdendritic Spaces

**Figure 16:**
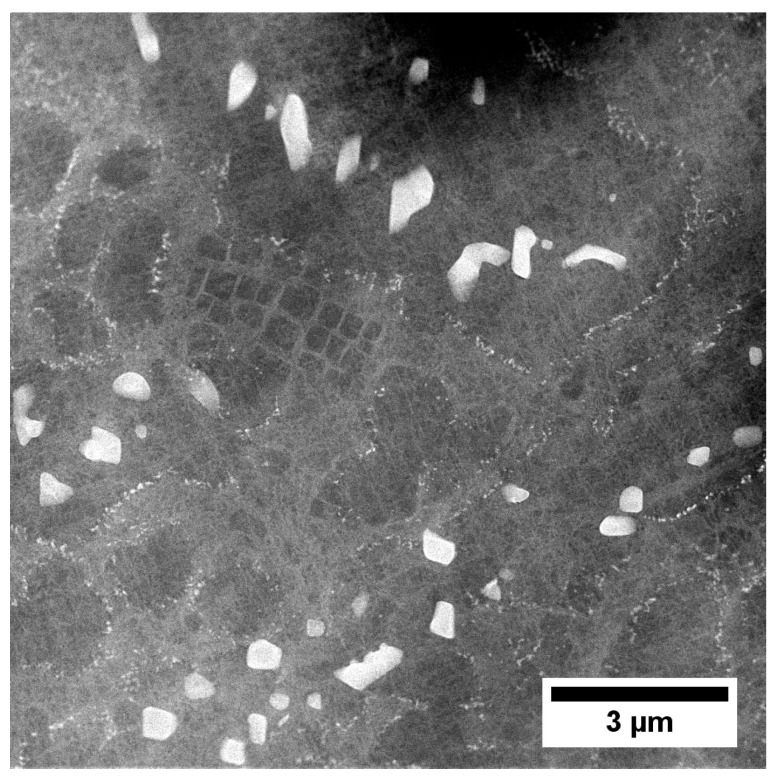
Microstructure of precipitates in the interdenritic spaces, STEM-HAADF.

Numerous Hf and Ta-rich carbides, as well as the γ-γ′ eutectic islands, were revealed during the SEM studies of interdendritic spaces. Further observations with transmission electron microscopy were done to show the remaining phases. The microstructure of the interdendritic spaces is much more complex than that of the dendritic regions. The difference is due both to the more complex morphology of the precipitates and their phase contrast. One similarity to the dendritic regions is numerous M_5_B_3_ borides at the γ/γ′ interfaces (Figure 16). It should be noted that boron may reduce the solubility of carbon in the γ matrix, which promotes carbides’ precipitation.

**Figure 17:**
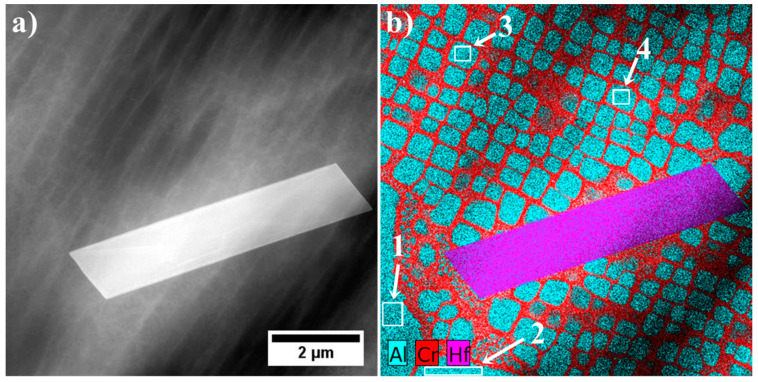
(**a**) location of mapping; (**b**) distribution of selected alloying elements in the area of primary γ′, secondary γ′, and MC carbide, STEM-EDX.

The chemical composition of primary and secondary (cubic) γ′ was investigated. The locations of mapping, distribution of selected alloying elements, and the areas subjected to quantitative analysis are shown in Figure 17 and Table 6. In areas no. 1 and 2 involving primary γ′ precipitates, the total content of γ′-formers (Al, Ti, Ta, Hf) is 15.5 at% and 16.1 at%, respectively. In the areas no. 3–4, which correspond to the secondary γ′ precipitates, the values are lower and ranged from 13.3–14.9 at%.

**Figure 18:**
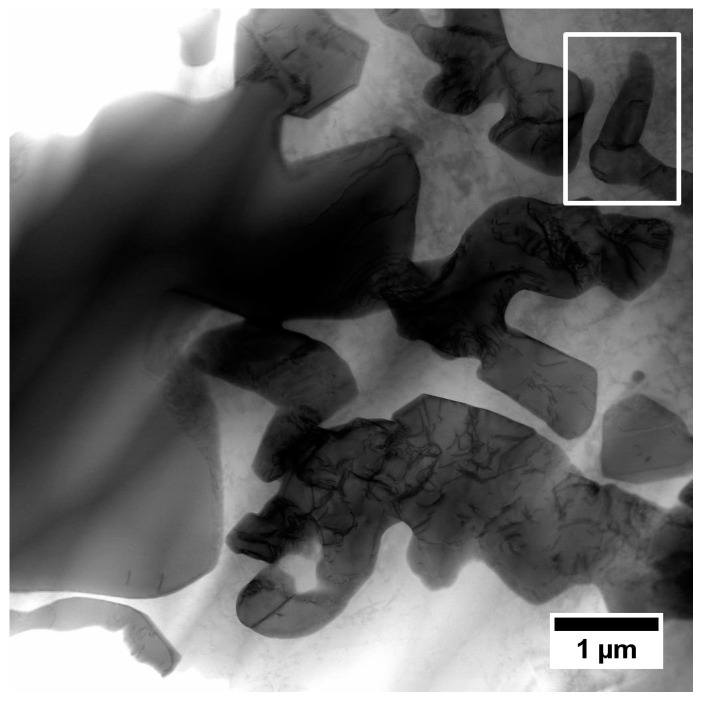
Microstructure of “Chinese script” carbide in interdendritic space, STEM-HAADF.

**Figure 19:**
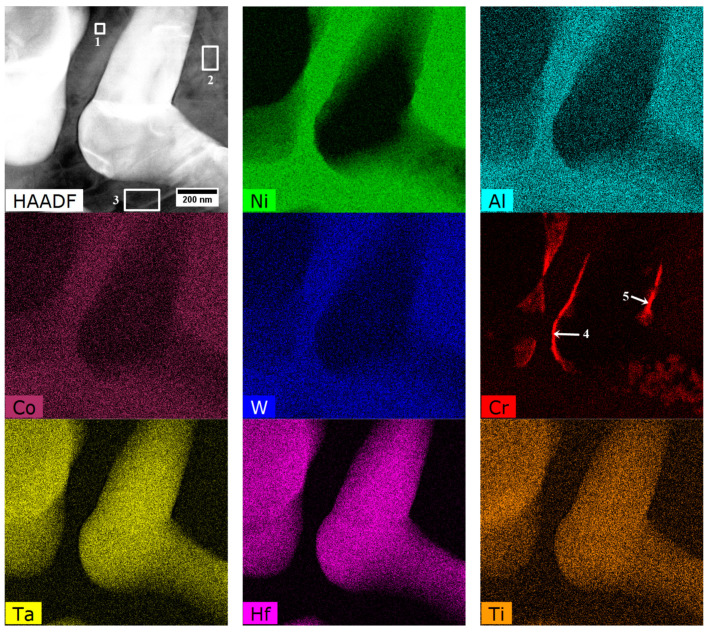
Distribution of selected alloying element in the fragment of “Chinese script” carbide, STEM-EDX.

STEM-EDX mapping was performed in the region which includes a fragment of the “Chinese script” carbide, surrounded by a coarse γ′ precipitate (Figure 18 and Figure 19). Additionally, three quantitative analyses of the coarse γ′ chemical composition were carried out. Based on the results in Table 7, the total content of Al, Ta, Ti, and Hf in the γ′ phase precipitates in an area no. 1–3 is 17.3 at%, 16.3 at%, and 16.9 at%, respectively. The Cr distribution revealed the nano-precipitates not observed by SEM. They are present locally on the edges of the MC carbides and are composed of mainly of Cr, whose concentration is clearly higher than of W and Mo. Thus, the Cr/W relation in region no. 4 and 5 is 21.9 and 13.3, respectively. It should be noted that similar layers enriched in Cr were not found on the edge of blocky carbides near eutectic γ-γ′ islands, which indicates differences in thermodynamical stability at elevated temperatures. 

**Figure 20:**
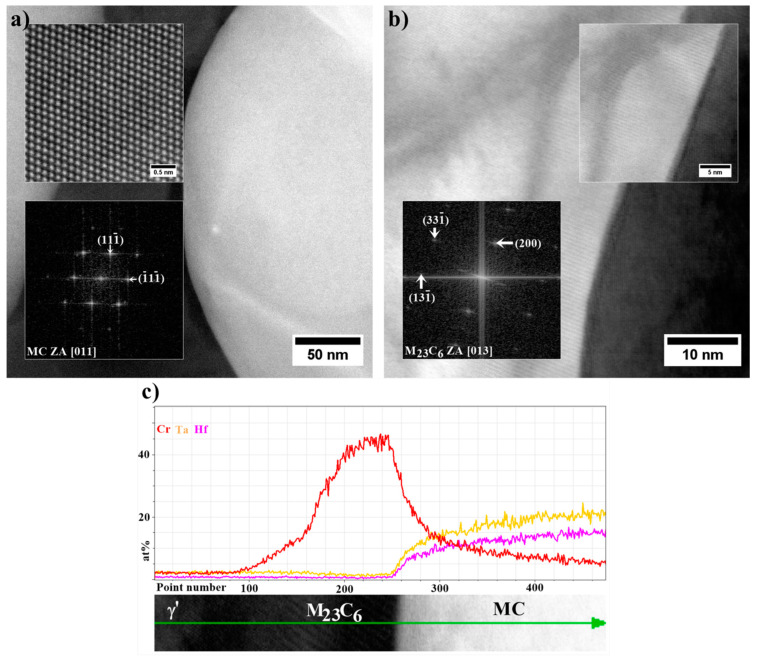
(**a**) atomic structure and FFT image of MC carbide (STEM-HAADF); (**b**) the nano-precipitate of M_23_C_6_ at the MC edge (STEM-BF); (**c**) linear distribution of Cr, Hf and Ta (STEM-EDX).

Locally on the edges of MC carbides, nano-layers with width 5–15 nm are present (Figure 20a,b). Reflections assigned to the FFT image correspond to the M_23_C_6_ carbide, which possesses a cubic crystal structure composed of 116 atoms (D8_4_ type). In order to reveal the distribution of selected elements at the M_23_C_6_/γ′ and M_23_C_6_/MC interfaces, additional linear measurements were performed (Figure 20c). The Cr smooth distribution changes at the interfaces γ′/M_23_C_6_ and M_23_C_6_/MC, indicating that the presence of M_23_C_6_ carbides can be explained by the interaction between the MC carbide and the matrix during the heat treatment.

M_23_C_6_ carbides are formed in medium- and high-chromium superalloys during casting’s crystallization, heat treatment or annealing in the range of 760-980 °C. The alloying elements replacing chromium in the crystal lattice could increase the lattice parameter [8]. The position of M is mainly occupied by Cr. However, numerous alloying elements, including Mo and W, lead to a much more complex composition, e.g., Cr_21_(Mo, W)_2_C_6_ and (Ni, Co, Fe, Cr)_21_(Mo, W)_2_C_6_. In superalloys with increased iron content, it can be expected that they will partially replace chromium. In the solid-state, the M_23_C_6_ carbides are precipitating directly from the carbon-enriched γ matrix or by the decomposition reaction of the MC carbide, usually along grain boundaries, at twin boundaries, and on stacking faults [37,43]. Long-term service or heat treatment of superalloys at elevated temperatures may lead to the decomposition of MC carbides into more stable M_23_C_6_ and M_6_C with lower carbon content, according to the following reactions [23]:

MC + γ → M_23_C_6_ + γ′, (I)

MC + γ → M_23_C_6_ + η, (II)

MC + γ → M_6_C + γ′, (III)

The similarity in crystal structure sometimes makes the M_6_C and M_23_C_6_ carbides challenging to distinguish. The M_6_C usually replace M_23_C_6_ carbides in alloys containing approximately over 6 wt% Mo plus its atomic equivalent in W (weight per cent Mo plus one half the weight per cent W) [9,44,45]. The combined HRSTEM and STEM-EDX analyses show that I-type phase transformation occurred in René 108 during heat-treatment. The same transformation was also observed in crept Inconel 713C superalloy at 982 °C [46,47]. The Cr-rich M_23_C_6_ carbides then precipitated along the grain boundaries in the form of blocks surrounded by the γ′ precipitates. The degradation of the microstructure after annealing of the GTD111 superalloy at lower temperatures, 927 °C and 871 °C, mainly included the type II transformation [48]. The η phase (tetragonal structure with a = b = 5.09 Å, c = 8.29 Å) was particularly favoured by the enrichment of MC carbides in Ti and Ta because the Ti/Al concentration ratio in the superalloy exceeded 1.5. Relatively high Ti/Al ratio in superalloys promotes η phase formation, which, due to significant difference in lattice parameter compared to γ matrix, sensitizes the η/γ interface to crack nucleation and consequently decreases creep rupture life [42,49,50]. In this study, the Ti/Al relationship is 0.125, which is not favourable for the II-type phase transformation. The last possible phase transformation includes the formation of M_6_C. The nano M_6_C carbides discontinuously precipitated in the γ matrix or along γ/γ′ interfaces of the crept K416B superalloy (1100 °C) were found by Xie [51]. Based on the thermodynamic analyses, solubility of C in the γ phase decreases during creep, which leads to its segregation in the stress concentration areas and then combining with carbide-formers such as W.

**Figure 21:**
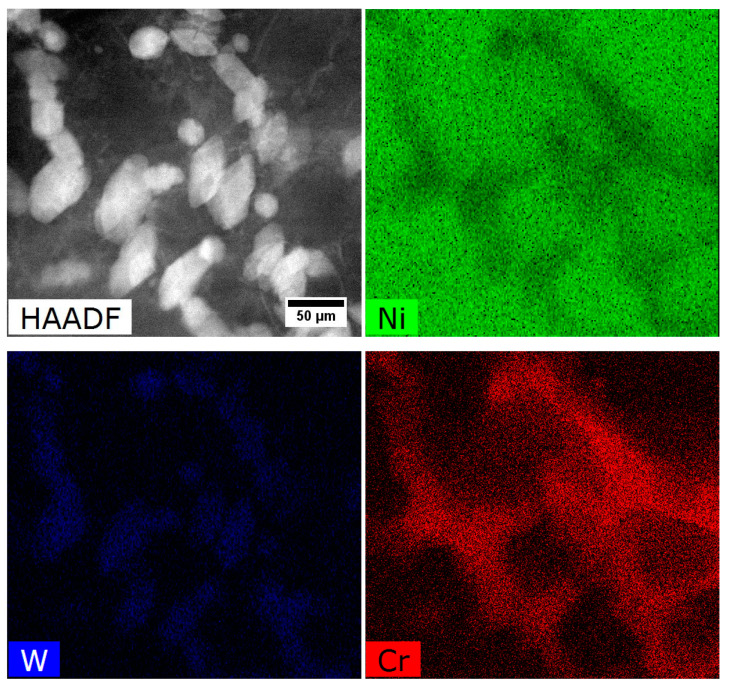
Distribution of selected alloying elements at a grain boundary, STEM-EDX.

**Figure 22:**
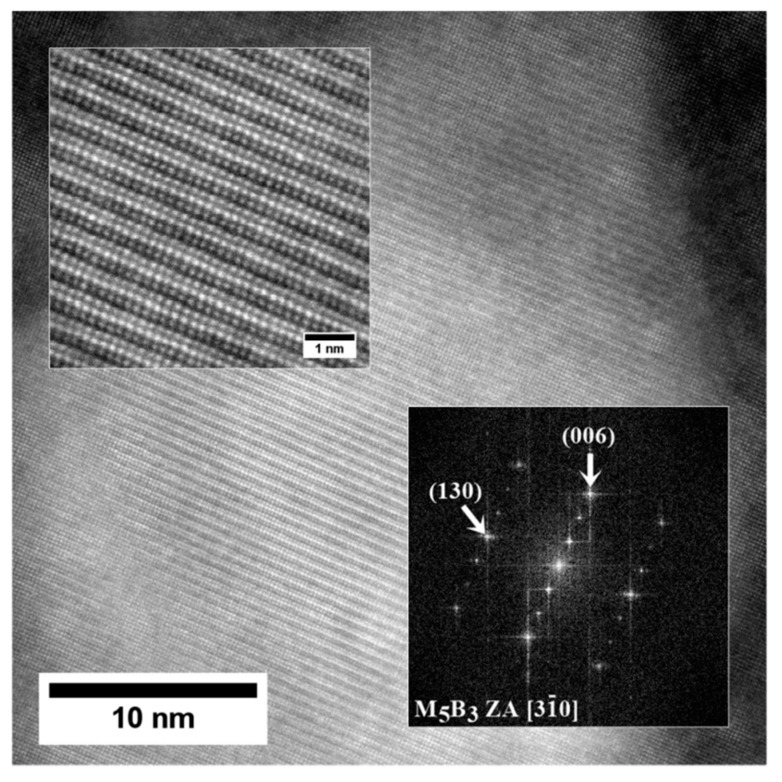
Nanostructure of the M_5_B_3_ boride located at grain boundary, HRSTEM-HAADF.

During the SEM observation, precipitates with a clear and bright contrast (high Z-number) are revealed along the grain boundaries. This area has also been subjected to detailed STEM and HRSTEM studies. Figure 21 shows the microstructure of the precipitates along the grain boundary and the distribution of Ni, W and Cr in this area. The morphology and size of these precipitates are the same as these at the γ/γ′ interfaces. The second similarity is enrichment in the same alloying elements. Their nanostructure with the corresponding FFT image is presented in Figure 22. The M_5_B_3_ borides are confirmed. Imaging this phase using tilting of grain boundary region revealed numerous precipitates. On the SEM-BSE, such precipitates’ agglomeration is shown as a continuous or semi-continuous layer.

It has been shown that the addition of boron to Ni-based superalloys could significantly influence the grain boundary precipitation [31,52,53]. B atoms are larger than those of the other interstitial atoms and smaller than those of the substitutional atoms in Ni-based superalloys. So, the lattice distortion caused by boron is considerable. The small diameter of boron allows it to fill vacancies at the grain boundaries, reducing the diffusivity in these regions [54]. During the ageing-treatment, boron segregation to the grain boundaries occurs and a large number of solute atoms, such as tungsten, chromium, and molybdenum, distribute at the grain boundaries. Because W, Cr, and Mo are more inclined to dissolve in the γ matrix when intermetallic γ′ phase is formed, these elements would be expelled and gather at the γ/γ′ interfaces. As a result, B would interact with W, Cr, and Mo and form M_5_B_3_ borides. Their formation at grain boundaries would pin these boundaries and impede grain-boundary sliding mechanism at high-temperatures. Xiao suggested that any strong interaction between B atoms and dislocation cores could hinder dislocation motion and thus increase resistance to fatigue failure [55]. 

## 4. Conclusions

- The FCC γ matrix is strengthened mainly by coherent precipitates characterised by ordered L1_2_ crystal structure. The mean misfit coefficient between matrix and precipitates is δ = +0.6%.
- Dendritic structure with significant segregation of alloying elements and microstructural constituents is observed.
- The mean volume fraction of MC carbides is around 0.8% (LM), while γ′ precipitates in dendritic regions around 54.99% (SEM-BSE).
- In dendritic regions the γ′ precipitates have a complex morphology with two classes of shape factor for which the mean ξ values are 0.39 and 0.76. Too high diversity in size and morphology of precipitates in interdendritic spaces did not allow their effective comparison.
- The MC carbides are preferentially precipitated in the interdendritic areas. The “M” position is occupied mainly by Ta, Hf, Ti, and W, while the mutual concentration relationships depend on the morphology.
- The MC carbide degradation occurred during ageing according to the phase transformation reaction: MC + γ → M_23_C_6_ + γ′. The M_23_C_6_ carbides are revealed at the MC edges as nano-layers with width 5-15 nm.
- The M_5_B_3_ borides characterised by body-centered tetragonal I4/mcm crystal structure have polygon and rhombus forms in thin foils. They are preferentially formed at the interfaces of secondary γ′ with matrix as nano-precipitates, both in the dendritic and interdendritic regions, and also on the grain boundaries. The STEM-EDX analysis revealed that they are enriched mainly in W, Cr, and Mo.

## Figures and Tables

**Table 1:** Chemical composition of René 108 determined by optical emission spectroscopy (OES), wt%.

Element	Cr	W	Co	Al	Ta	Hf	Ti	Mo	C	B	Zr	Ni
Concentration	11.4	8.9	8.2	6.4	3.6	1.5	0.8	0.5	0.08	0.02	0.0125	Bal.

**Table 2:** Solubility of selected alloying elements in nickel at 1200 °C and 900 °C. Calculated by Thermo-Calc.

Element	Radius, Å	Solubility in Nickel, wt%/at%	
		1200 °C	900 °C
Ni	1.49		
Cr	1.66	46.50/49.53	38.49/41.40
Co	1.52	100/100	100/100
W	1.93	39.01/16.96	35.31/14.84
Al	1.18	9.38/18.38	7.17/14.38
Ta	2.00	20.19/7.58	12.87/4.57
Hf	2.08	3.67/1.24	0.78/0.26
Ti	1.76	11.98/14.30	8.58/10.32
Mo	1.90	36.45/25.97	27.77/19.04
B	0.87	0.067/0.36	0.019/0.10
C	0.67	0.426/2.05	0.162/0.79
Zr	2.06	1.047/0.68	0.591/0.38

**Table 3:** The measured values of d_hkl_ spacing and lattice parameters for γ, γʹ, MC carbides in the René 108.

(hkl)	d_hkl_, Å	a, Å
	γ matrix FCC Fm$\bar{3}$m (225): PDF No. 47-1417	
(111)	2.07	3.59
(200)	1.80	3.60
(220)	1.27	3.58
(311)	1.08	3.58
(222)	1.04	3.59
	γ′ FCC ordered L1_2_: PDF No. 65-3245	
(111)	2.09	3.62
(200)	1.81	3.63
(210)	1.60	3.58
(220)	1.27	3.59
(221)	1.20	3.60
(311)	1.08	3.60
(222)	1.04	3.60
	MC FCC Fm$\bar{3}$m (225): PDF No. 74-1223	
(111)	2.61	4.52
(200)	2.26	4.52

**Table 4:** Results of SEM-EDX analysis in MC carbides, at%.

No.	Ta	Hf	Ti	Ni	W	Co	Cr	Mo	Ta/Hf	(Ta + Hf)/(W + Ti)	(Ta/Hf)/(W + Ti)
1	12.2	75.0	1.1	6.9	1.0	1.4	1.0	1.4	0.16	42.95	0.08
2	16.3	73.3	1.2	5.4	1.2	1.0	0.9	0.7	0.22	37.99	0.09
3	16.6	72.3	2.0	6.0	0.8	0.7	1.1	0.5	0.23	32.32	0.08
4	42.5	21.1	17.4	7.7	5.9	2.4	1.8	1.3	2.01	2.73	0.09
5	42.7	16.8	19.1	8.2	7.1	2.4	2.3	1.4	2.54	2.27	0.10
6	43.9	19.9	19.5	6.9	5.5	0.9	1.6	1.8	2.20	2.56	0.09
7	42.8	18.3	18.4	7.9	7.0	2.2	1.8	1.7	2.35	2.40	0.09
8	23.4	54.5	6.5	5.7	5.9	1.4	1.0	1.6	0.43	6.26	0.03
9	17.5	61.1	4.2	5.9	5.5	2.9	2.1	1.0	0.29	8.06	0.03
10	44.9	21.5	18.9	5.9	5.2	1.1	1.0	1.5	2.09	2.76	0.09
11	30.9	41.3	10.0	6.9	5.2	2.6	2.0	1.1	0.75	4.75	0.05
12	20.2	19.9	6.4	5.6	1.1	1.9	1.6	20.2	2.15	2.49	0.08

**Table 5:** Chemical composition of the precipitates in dendritic regions, STEM-EDX, at%.

Area	Phase	Ni	Cr	Co	W	Al	Ta	Hf	Ti	Mo
1	Matrix γ	52.1	21.5	16.9	4.7	3.4	0.3	0.2	0.2	0.7
2	Secondary γ′	69.8	2.9	6.8	3.2	13.7	1.6	0.6	1.1	0.3
3	Tertiary γ′	69.4	2.8	7.3	4.8	13.4	0.9	0.3	0.8	0.5
4	Boride M_5_B_3_	10.0	35.5	3.2	42.0	2.3	1.2	0.3	0.4	5.2
5		50.9	12.4	5.9	17.1	9.4	1.3	0.4	0.7	1.9

**Table 6:** Chemical composition of the primary and secondary γ′ precipitates in interdendritic space, STEM-EDX, at%.

Area	Phase	Ni	Cr	Co	W	Al	Ta	Hf	Ti	Mo
1	Primary γ′	71.0	3.5	7.1	2.6	11.6	1.4	1.1	1.4	0.3
2		70.2	3.6	7.0	2.8	12.3	1.5	1.0	1.3	0.3
3	Secondary γ′	71.8	2.7	6.5	2.7	11.2	1.3	1.0	1.4	0.6
4		74.1	2.7	6.5	3.1	10.0	1.3	0.9	1.1	0.5

**Table 7:** Chemical composition of the coarse secondary γ′ and M_23_C_6_ (at the MC carbide edge), at%.

Area	Phase	Cr	Co	W	Al	Hf	Ta	Ti	Mo	Ni
1	Coarse secondary γ′	3.0	7.5	3.3	11.8	1.1	2.7	1.7	0.5	68.4
2		3.5	7.3	2.7	10.9	1.0	2.6	1.8	0.8	69.6
3		3.4	7.3	2.9	11.5	0.9	2.7	1.8	0.6	68.8
4	M_23_C_6_	43.7	4.5	2.0	6.2	3.3	6.5	2.7	1.9	29.4
5		53.3	6.3	4.0	2.5	2.8	5.3	1.6	2.2	22.0
